# Numerical modelling of micron particle inhalation in a realistic nasal airway with pediatric adenoid hypertrophy: A virtual comparison between pre- and postoperative models

**DOI:** 10.3389/fped.2023.1083699

**Published:** 2023-02-23

**Authors:** Qinyuan Sun, Jingliang Dong, Ya Zhang, Lin Tian, Jiyuan Tu

**Affiliations:** ^1^ School of Engineering, RMIT University, Bundoora, VIC, Australia; ^2^ Department of Otolaryngology Head and Neck Surgery, The Second Affiliated Hospital of Xi’an Jiaotong University, Xi’an, China

**Keywords:** nasal airway, numerical simulation, adenoid hypertrophy, obstruction, aerosol transport, olfactory

## Abstract

Adenoid hypertrophy (AH) is an obstructive condition due to enlarged adenoids, causing mouth breathing, nasal blockage, snoring and/or restless sleep. While reliable diagnostic techniques, such as lateral soft tissue x-ray imaging or flexible nasopharyngoscopy, have been widely adopted in general practice, the actual impact of airway obstruction on nasal airflow and inhalation exposure to drug aerosols remains largely unknown. In this study, the effects of adenoid hypertrophy on airflow and micron particle inhalation exposure characteristics were analysed by virtually comparing pre- and postoperative models based on a realistic 3-year-old nasal airway with AH. More specifically, detailed comparison focused on anatomical shape variations, overall airflow and olfactory ventilation, associated particle deposition in overall and local regions were conducted. Our results indicate that the enlarged adenoid tissue can significantly alter the airflow fields. By virtually removing the enlarged tissue and restoring the airway, peak velocity and wall shear stress were restored, and olfactory ventilation was considerably improved (with a 16∼63% improvement in terms of local ventilation speed). Furthermore, particle deposition results revealed that nasal airway with AH exhibits higher particle filtration tendency with densely packed deposition hot spots being observed along the floor region and enlarged adenoid tissue area. While for the postoperative model, the deposition curve was shifted to the right. The local deposition efficiency results demonstrated that more particles with larger inertia can be delivered to the targeted affected area following Adenoidectomy (Adenoid Removal). Research findings are expected to provide scientific evidence for adenoidectomy planning and aerosol therapy following Adenoidectomy, which can substantially improve present clinical treatment outcomes.

## Introduction

As the first organ of human respiratory system from the external environment, nasal cavity plays an essential role for humidifying and conditioning the inhaled air to nearly alveolar conditions in order to prevent potential risks from delicate lungs (1). The internal structures of the nose also filter airborne particulate which is conveyed towards the mouth by mucociliary clearance (2–4). On the other hand, intranasal drug delivery has been employed as a major route of mucosal administration for local treatment of allergic disease, nasal congestions or infections due to the abundant supply of vasculature in nasal cavities (5–7). For current view, anatomy and physiology of nasal cavity is a key factor that influencing the inhaled airflow patterns so that nasal disorder does affect the performance of its functions (8–10). Deepening the understanding of nasal airflow dynamics will help guide the evaluation and improvement of nasal airway obstruction in related fields, especially in clinical practice.

Numerous studies have explored approaches of measuring nasal airflow parameters by in-vitro experiments (11–14). Gradually, compared with in-vitro replica experiment, researches trend to using computational fluid dynamics(CFD) method since its provides more accurate and detailed prospect of presenting complex and variable airflow patterns (15–18). Compared with in-vitro studies, numerical simulation by computational fluid dynamics(CFD) method present significant advantages of providing rich flow patterns and inhalable particles transport. However, most of these studies conducted among adult group while children's underdeveloped nasal passages that compared are lack of investigation. Xi, Si (19) evaluated the transport and deposition of micro-particles (in spectrum of 0.5–32 μm) in a nasal-laryngeal airway model based on MRI images of a 5-year-old boy. This study emphasized the child-adult difference in nasal physiology and deposition efficiency. Based on the result, much higher breathing resistance was observed in the child model. Also, deposition patterns were sensitive to inhalation flow rate under low activity conditions. Later, Xi, Berlinski (20) evaluated age-related effects on airflow and aerosol dynamics in multiple MRI image based nasal-laryngeal airway subjects of a 10-day-old newborn, a 7-month-old infant, a 5-year-old child and a 53-year-old adult. Despite the significantly different airway morphology and dimension, results indicate that the total deposition fractions exhibit similar variation trends for ultrafine aerosols (1–100 nm). In contrast, for localised deposition patterns in the sub-region (i.e., turbinate, nasopharynx, larynx) are quite different among the four age groups. Further, their another study investigated micrometre particles (2.5–40 μm) transport based on the same models and reported substantial variation of regional deposition in vestibule, turbinate and nasopharynx (21). Besides, Moreddu, Meister (22) evaluated nasal resistance of five children subjects aged from 8 to 15 years based on active anterior rhinomanometry (AAR). The obstruction levels were also assessed *via* numerical simulations based on 3D models reconstructed from CT scans. Their results revealed that numerically predicted trans-nasal pressure drop in these five models achieved great consistency with the clinical examination. Moreover, Su, Lee (23) examined nasal airflow of a five-year-old Malaysian child model that reconstructed from CT scans. They found that flow field in the child's nasal cavity child tends to concentrate centrally.

Though multiple approaches were adopted in previous work in order to enrich understanding of airflow dynamics characteristic in healthy children upper airway, still relatively limited methods were taken for disorder nasal cavity especially in children. Several studies have reported common nasal disorders in children determined through clinical measurement means, such as nasal septum deviation, turbinate hypertrophy, etc (24–26). Especially, adenoid hypertrophy (AH) commonly reported in childhood which observed as adenoid tissue abnormally enlarged (27). The adenoid is defined as an aggregation of lymphoid tissue located in the superior and posterior wall of the nasopharynx. The diseased adenoid tissue. Obstructive adenoids may cause nasal obstruction, mouth breathing, snoring, rhinorrhea, postnasal drip, cough, dry mouth, halitosis, swallowing difficulty, hypo-nasal voice, restlessness sleep, enuresis, and morning headache. In severe cases, they may induce obstructive sleep apnea, otitis media with effusion, and craniofacial growth abnormality, where Adenoidectomies are commonly considered a definitive treatment (28, 29). Considering the reliability and effectiveness of the surgery, nowadays, CFD has become a popular method for surgery prediction because of its excellent capacity of nasal airflow changes analysis. Clipp, Vicory (30) performed virtual surgery on the upper airway model which observed with symptom of difficult breathing and assess post-surgery flow characteristic to provide near real-time feedback to the clinician. Otherwise, a study in 2020 established an average geometry benchmark of 47 healthy nasal cavities and compared with the disorder nasal cavity diagnosed with nasal airway obstruction (NAO) as a reference (31). A virtual septoplasty was employed on the disorder model to measure the minimal cross-section area and nasal resistance, with the purpose of providing further background support for improving septoplasty outcomes in future study. Though virtual surgery prediction shows great prospects, most of the study employed adult samples that children characteristics are ignored. To make up the gaps, this study carried out based on the child nasal geometry to provide nasal airflow pre-operative assessment and post-operative care. This research aims to supplying scientific evidence to enable detailed examination of the disease and improve treatment outcomes.

## Method

### Model development of pre- and post-operative nasal airways

In this study, a nasal airway of a 3-year-old patient who was diagnosed with adenoid hypertrophy (AH) was reconstructed from 269 CT images (DICOM format) with a resolution of 512 × 512 pixels and 0.5 mm intervals. The set of clinical images were taken at the end of inhalation processes and the patient was required to hold breath during scanning, which provides a clear vision of original nasal airway. The imaging data was collected by project members in Xi'an Jiaotong University, which was conducted with written informed consent from the parents and was approved by the Human Research Ethics Committee at the Second Affiliated Hospital of Xi'an Jiaotong University.To facilitate the computational simulation application, the set of medical imaging data was imported into 3D-slicer version 5.0.3 [open-source platform (available http://www.slicer.org)] for nasal geometry segmentation and registration purpose. Detailed reconstruction process has been reported in our previous work (32).

To better restore the flow patterns and particle transport behaviors which were jointly influenced by the external nares and surrounding environment, the realistic facial features around nares that enclosed by a spherical breathing zone has been attached to the nasal passage (33). The larynx region was preserved to obtain the fully developed outstream and better numerical convergence.

**Figure 1:**
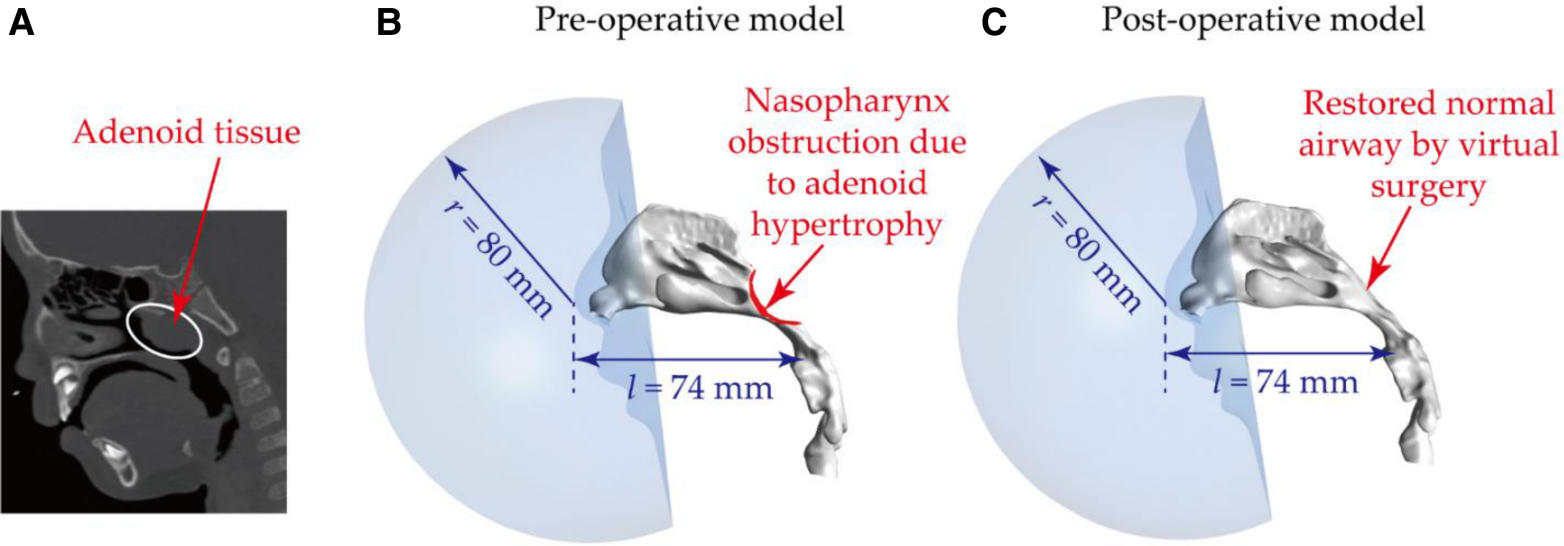
Nasal airway models based on obstructed nasal airway: (**A**) CT image showing the presence of the abnormal adenoid tissue – adenoid hypertrophy; (**B**) reconstructed nasal airway model with nasopharynx obstruction – pre-operative model; (**C**) restored normal nasal airway after virtual adenectomy – post-operative model. Note, to produce realistic flow fields and exposure conditions, a spherical breathing region with a radius of 80 mm was added to enclose the nares and external nose region.

To facility visualized the influence of adenoidectomy on nasal flow patterns and aerosols transport, a virtual surgery was conducted on the adenoid region and removed the enlarged adenoid tissue artificially as shown in Figure 1. Except for the artificially restored adenoid region, the post-operative geometry keeps consistence with the pre-operative geometry.

### Mesh configuration

**Figure 2:**
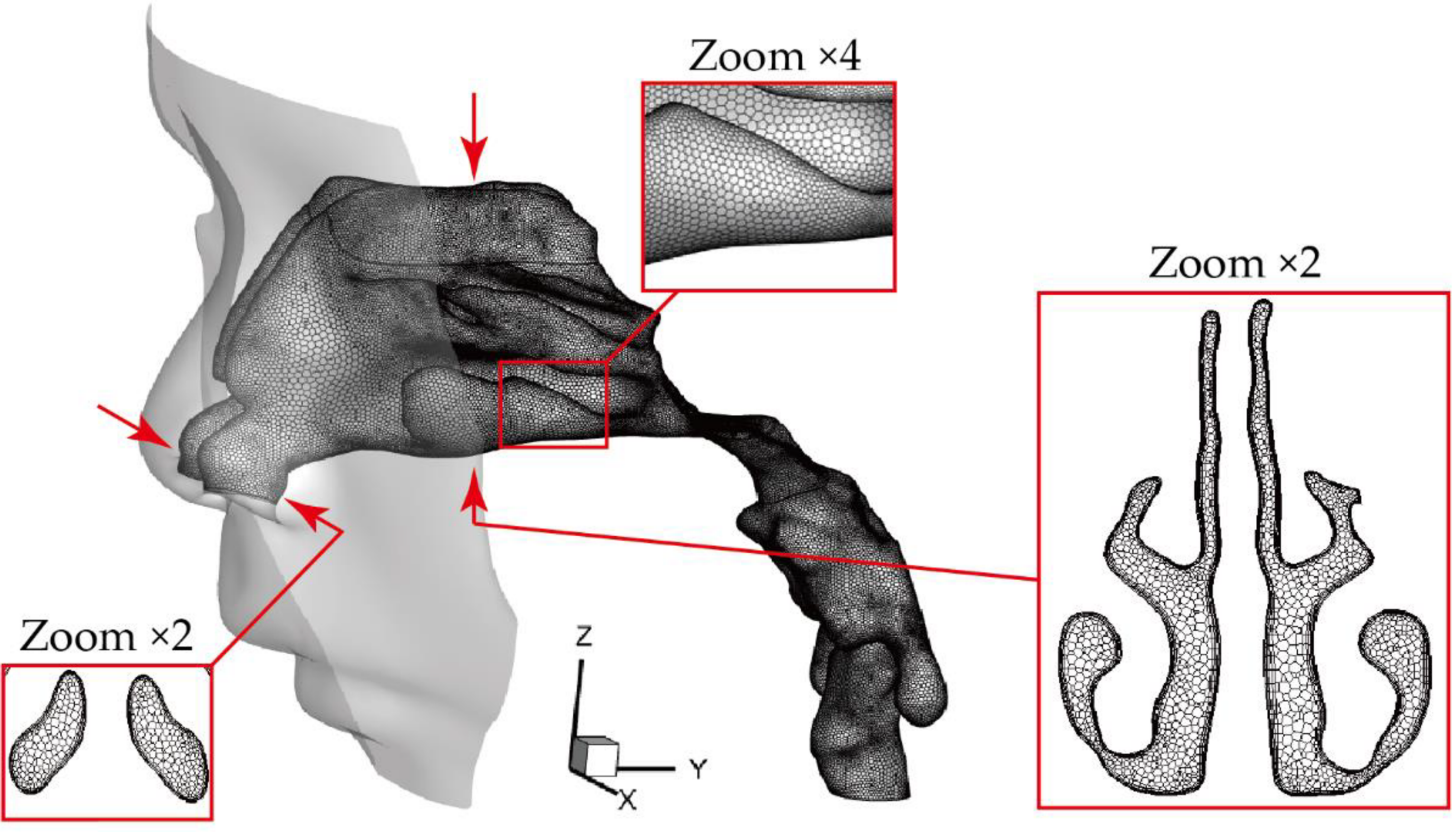
Preview of computational mesh (polyhedral mesh and prism mesh).

Figure 2 is a configuration preview of mesh details with two typical slices respectively located at nostrils and middle of the passage to highlight the internal mesh refinement. In each model, ANSYS Fluent Meshing (ANSYS, INC., CANONSBURG, Pennsylvania) was used for mesh generation, where polyhedral elements were employed to solve the flow fields. Compared with unstructured tetrahedral element, polyhedron scheme performs several advantages including less sensitive to stretching, better numerical convergence and more effective computational time. Meanwhile, 5 prism layers were attached to nasal walls and the face to provide an accurate prediction of particle behaviour in the near wall region. Subsequently, a mesh independence test was conducted by solving the average velocity at vestibule region under the inhalation flow rate of 9.5 LPM, where a stable solution was achieved when the global elements in main nasal cavity sized between 0.2 mm to 0.3 mm. Finally, 0.96 million and 1.19 million elements were used respectively for pre- and post-operative model, which achieved a balance between computational efficiency and accuracy. Overall, both pre- and post-operative model employed a hybrid prism-polyhedral mesh scheme to accomplish numerical accuracy while minimize computation cost.

### Boundary conditions

**Figure 3:**
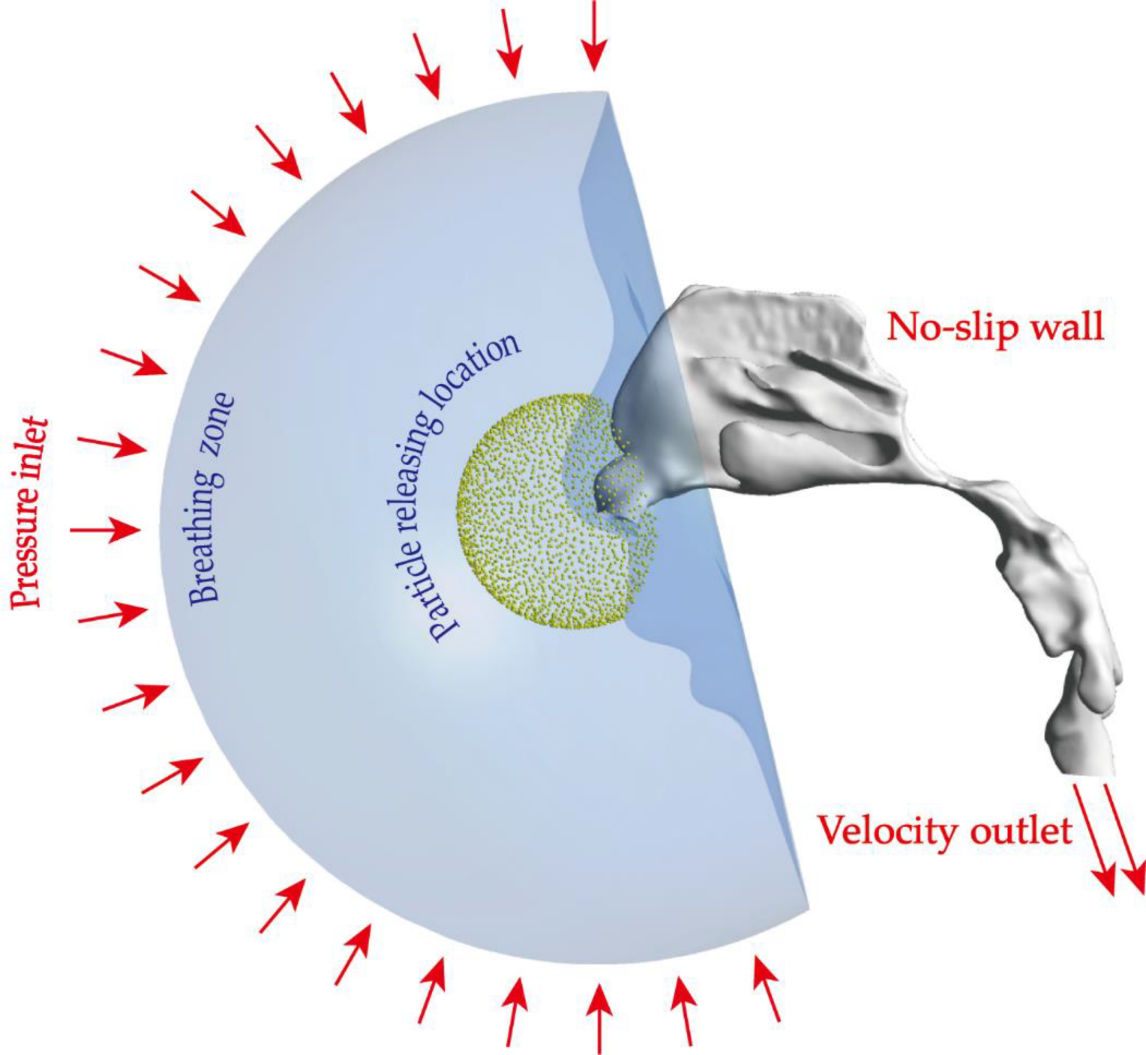
Setup of boundary conditions: pressure inlet condition was imposed on the hemispherical surface of the breathing zone, all nasal walls were set as no-slip condition, velocity outlet conditions were used to produce different flow fields. Micron particles were virtually released from a spherical location in front of the openings of nostrils.

Figure 3 illustrated the details of boundary conditions in this case. The attached breathing zone in front of the cavity was set as zero-gauge pressure inlet to imitate the ambient environment. The outlet of the airway was set as velocity outlet by dividing the physiological volumetric flow rate with the area of the outlet. International Commission on Radiological Protection (ICRP) publication 66 suggests a tidal volume of 0.244 L with a frequency of 39 per minute for children subjects under light exercise condition. Therefore, the equivalent volumetric flow rate of 9.5 litre per minute (LPM) was employed for light exercise condition. Respiratory conditions are determined by human exercise and physical activity. To consider different physiological conditions, numerical simulations under three levels of inhalation flow rates, representing, respectively, resting (3.1 LPM), light exercise (9.5 LPM), heavy exercise (18.9 LPM) circumstances were performed in this study. For the nasal surface as well as surrounding face, it was assumed to be no-slip, stationary and perfect absorbed when predict particle transport.

### Fluid flow simulation

In this study, the fluid flow was simulated by ANSYS-FLUENT 2020 R2 (ANSYS, INC., CANONSBURG, Pennsylvania). During quiet and moderate breathing, airflow is primarily laminar in nasal passages. However, the airflow may exhibit higher level of disturbances at locations with drastic changes in cross-sectional areas (such as the nasal valve or nasopharynx sections). Therefore, to capture all flow patterns in nasal airways including the laminar-turbulence transition, Shear-Stress Transport (SST) *k-ω* model was employed in present study (34, 35). Due to the presence of swirls and eddies, stronger mixing effect are expected in turbulent flow, which causes higher flow resistance. In addition, flow fluctuations also cause particles to undergo changes in both magnitude and direction of their trajectories and to eventually deposit on airway walls. To account for the role of turbulence in deposition, the Discrete Random Walk (DRW) model can be added when conducting air-particle simulations using Discrete Phase Model (DPM). According to our predictions of the total particle deposition efficiencies for 1 μm, 5 μm and 10 μm particles with and without the DRW model, the largest value disparity is 0.32% for 1 μm particles. Therefore, the effect of turbulent mixing on deposition is considered as insignificant and negligible.

For the assuming steady incompressible fluid, continuity and momentum equations were solved to govern the fluid motion and shown as below:

(1) $$\frac{\partial }{\partial x_{i}}\left(\rho u_{i}\right)=0$$

(2) $$\rho u_{j}\frac{\partial u_{i}}{\partial x_{j}}=-\frac{\partial p}{\partial x_{i}}+\frac{\partial }{\partial x_{j}}\left[\mu \frac{\partial u_{i}}{\partial x_{j}}\right]$$

Where *ρ* represents density, u represents velocity and p represents pressure of the air.

### Particle simulation

Previous studies have revealed that there is an increasing trend in adopting medical nebulizer and Metered-Dose Inhaler (MDI) approaches in adenoid hypotrophy therapy, and the resultant drug aerosol size distribution is generally below 20 µm (36–40). Therefore, micrometer particles ranged between 1 and 20 μm were employed to evaluate the aerosols transport and deposition performance. Micron-particle sized 1, 2, 3, 4, 5, 6, 7, 8, 10, 15, 20 µm were tracked. For each particle size, 50,000 were passively released based on a series of particle number independence tests with a variation less than 0.1% for predicted of deposition efficiency. Particles were uniformly released from a spherical surface with a radius of 30 mm centered at the nose tip, which fully overlay the nostril and its surrounding area. The particles were released with a zero initial velocity and inhaled along the inhalation streamlines. Primary mechanisms of aerosol deposition in respiratory airways include inertial impaction, gravitational sedimentation, Brownian diffusion, and to a lesser extent, by turbulence, electrostatic precipitation, and interception (41). The relative contribution of these mechanisms is a function of the physical characteristics of the particles, the airway anatomy, and the physiological airflow patterns. Inertial impaction mainly occurs in the upper respiratory tract when there is a sudden change in the airflow direction, which causes large micron particles to deviate from the air streamlines as the inertia of the particles keeps them on their initial trajectories. For gravitational sedimentation, it results from the settling of the particles under that action of gravity. This mechanism is most efficient in the small airways and alveoli where the residence time is high and the travel distance of particles is small. The third main mechanism of deposition is Brownian diffusion, it results from the random motions of the particles caused by their collisions with gas molecules. Unlike impaction and sedimentation, deposition by Brownian diffusion increases with decreasing particle size and becomes the dominant mechanism for particles smaller than 0.5 µm. For aerosol particles (micron-particle in a range between 1 and 20 µm) considered in this study, inertial impaction is the dominant mechanism of deposition, as previous studies have demonstrated that the very short residence time in the nasal passage (smaller than 0.1 s) do not permit micron-particles to deposit by sedimentation (42).

In this case, one-way coupled Lagrangian discrete phase model (DPM) was used to predict the individual particle trajectories which occupies a low volume fraction, equating the particle inertia with drag force, gravity force and Brownian force:

(3) $$\frac{du_{i}^{P}}{dt}=F_{D}+F_{G}+F_{B}$$

where $u_{i}^{P}$ represents the particles velocity, $F_{D}$ is the drag force per unit particle mass described as:

(4) $$F_{D}=\frac{18\mu \left(u_{i}^{g}-u_{i}^{p}\right)}{C_{c}d_{p}^{2}\rho_{p}}$$

here *u* is the airflow velocity, μ is the air viscosity, $d_{P}$ is the particle diameter, $\rho_{P}$ is the particle density and $C_{c}$ is the Cunningham correction factor given by:

(5) $$C_{c}=1+\frac{2\lambda }{d_{P}}\left(1.257+0.4e^{\left(-\;\frac{1.1d_{p}}{2\lambda }\right)}\right)$$

here λ is the air molecular mean free path defined as 67 mm in this case.

$F_{B}$ is Brownian force defined as $\xi_{i}\surd \left(\left(\pi S_{o}\right)/\Delta t\right)$, where Δt is the particle integration time-step and $\xi_{i}$ is a zero-mean, unit-variance-independent Gaussian random numbers. $S_{o}$ is a spectral intensity function explained as:

(6) $$S_{o}=\frac{216vk_{B}T}{\pi^{2}\rho d_{p}^{5}{\left(\frac{\rho_{P}}{\rho }\right)}^{2}C_{c}}$$

Here v is the kinematic viscosity, $k_{B}$ is the Boltzmann constant, T is the Kelvin temperature of inhaled air set as 293 K in this case and $C_{c}$ is the Cunnigham correction factor.

Particles that absorbed by trap nasal surface were statistically process as particle deposition efficiency (particle number trapped by regional nasal wall divided by total number inhaled into nasal chamber).

### Model validation

**Figure 4:**
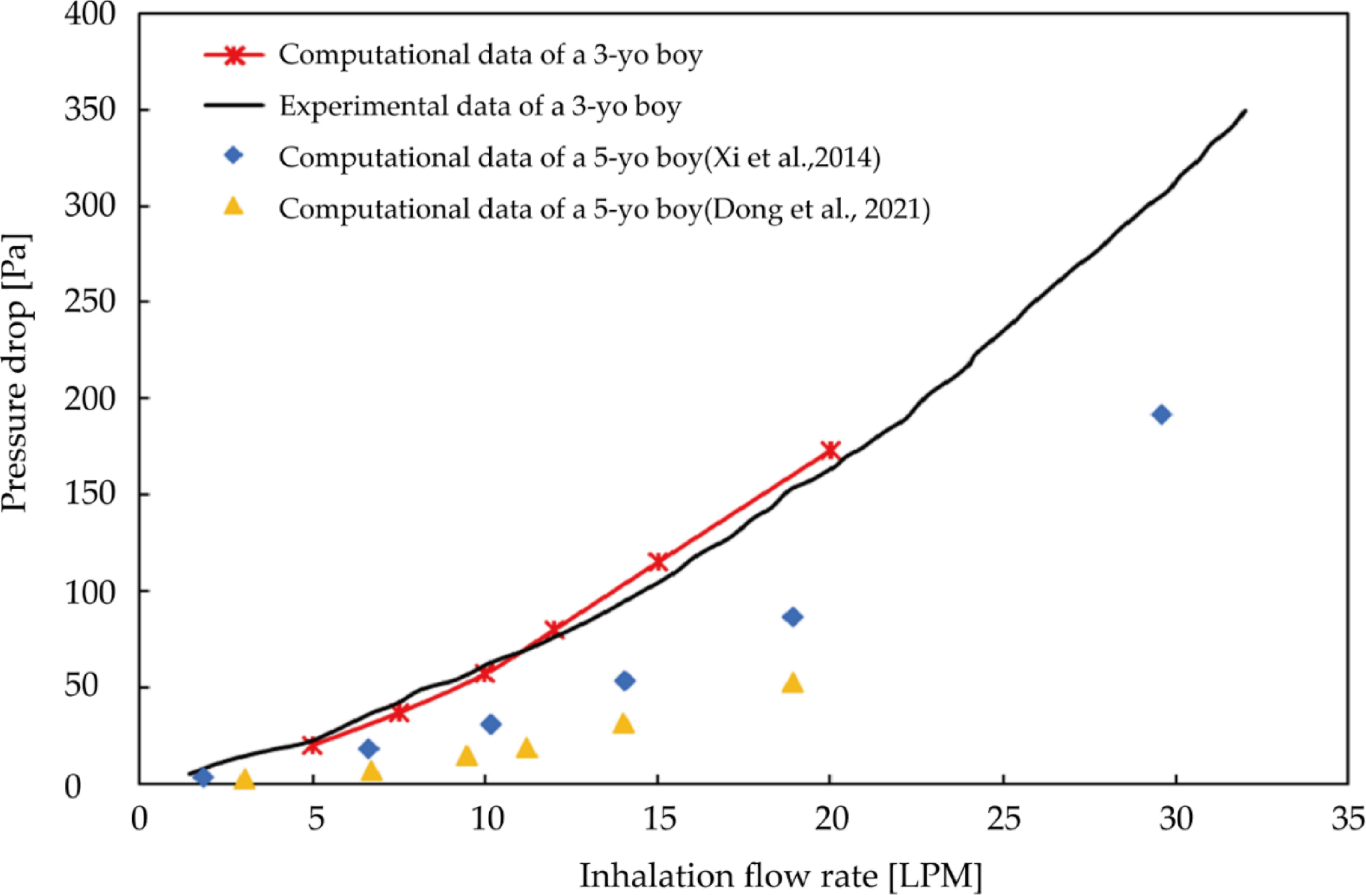
Measurement and numerical predicted trans-nasal pressure-flow curves with comparison data from previous literature.

To verify the reliability of the computational model, an in-vivo rhinomanometry measurement was conducted on the patient by project members in Xi'an Jiaotong University and the detailed measurement theory has been explained in our previous work (43). Figure 4 shows the measurement and numerical simulation results of the pressure drop-flow curves with comparison data from previous literature. Both of the prediction and measurement trans-nasal pressure drop trends to increase with the increasing inhalation flow rate. For a fixed flow rate, the numerical pressure-drop shows minimize tolerance with measurement results especially when flow rate is less than 10LPM while the difference slightly increases when inhalation at a higher rate. Overall, the numerical prediction curve shows great consistence with measurement curves both in magnitude and trend, which indicates convincing reliability of numerical model.

## Results and discussion

### Anatomical comparison of pre- and post-operation

**Figure 5:**
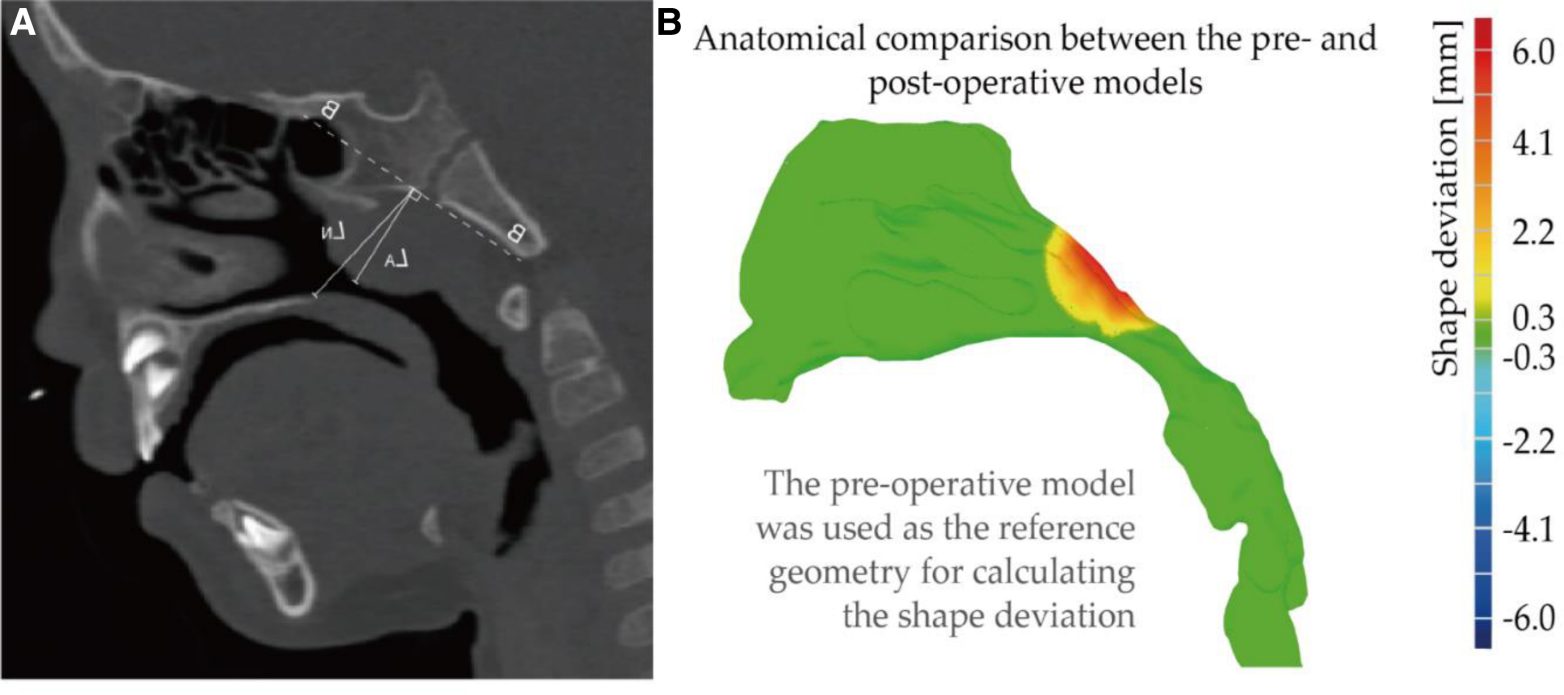
(**A**) illustrate of lateral neck x-ray(LNX) method (**B**) anatomical comparison between the pre- and post-operative models. Note, shape deviation was calculated when using the pre-operative model (original shape) as the reference geometry.

There are several clinical methods of adenotonsillar hypertrophy (AH) assessment to describe the degree of nasal airway obstruction. In this study, the lateral neck x-ray (LNX) was used for defining the degree, which is the most accepted method for assessing AH (44). Moideen, Mytheenkunju (45) reported that LNX assessment method has significant correlation with patient's symptoms and key landmarks of assessment according to the reference have been highlighted in the Figure 5A. Line B-B is the line drawn along straight part of anterior margin of basi-occiput, on which following measurement bases. Moideen defines the adenoid thickness (line L_A_) starts from line BB and then perpendicularly reaches most conves part of adenoid tissue. Nasopharynx depth (line L_N_) was measured from line BB to the spheno-occipital synchondrosis. Following Moideen's method, adenoid-to-nasopharyngeal ratio was then calculated as L_A_ divided by L_N_. In this study, 75% adenoid-to-nasopharyngeal ratio was measured, which belongs to Grade IV that enlargement tissue practically obstructs the airway.

Using original diseased model as reference location, shape deviation was investigated as Figure 5B, where red part indicates that adenoid tissue is reduced away from the airway after virtual surgery. The operation only performed virtual reduction of the enlarged tissue, and the blocked nasopharyngeal channel gradually widened that larger deformation was observed at the center of the adenoid pad. Relatively mild change about 1–2 mm was measured at the edge of the tissue, and the peak change near the center reached about 6 mm.

**Figure 6:**
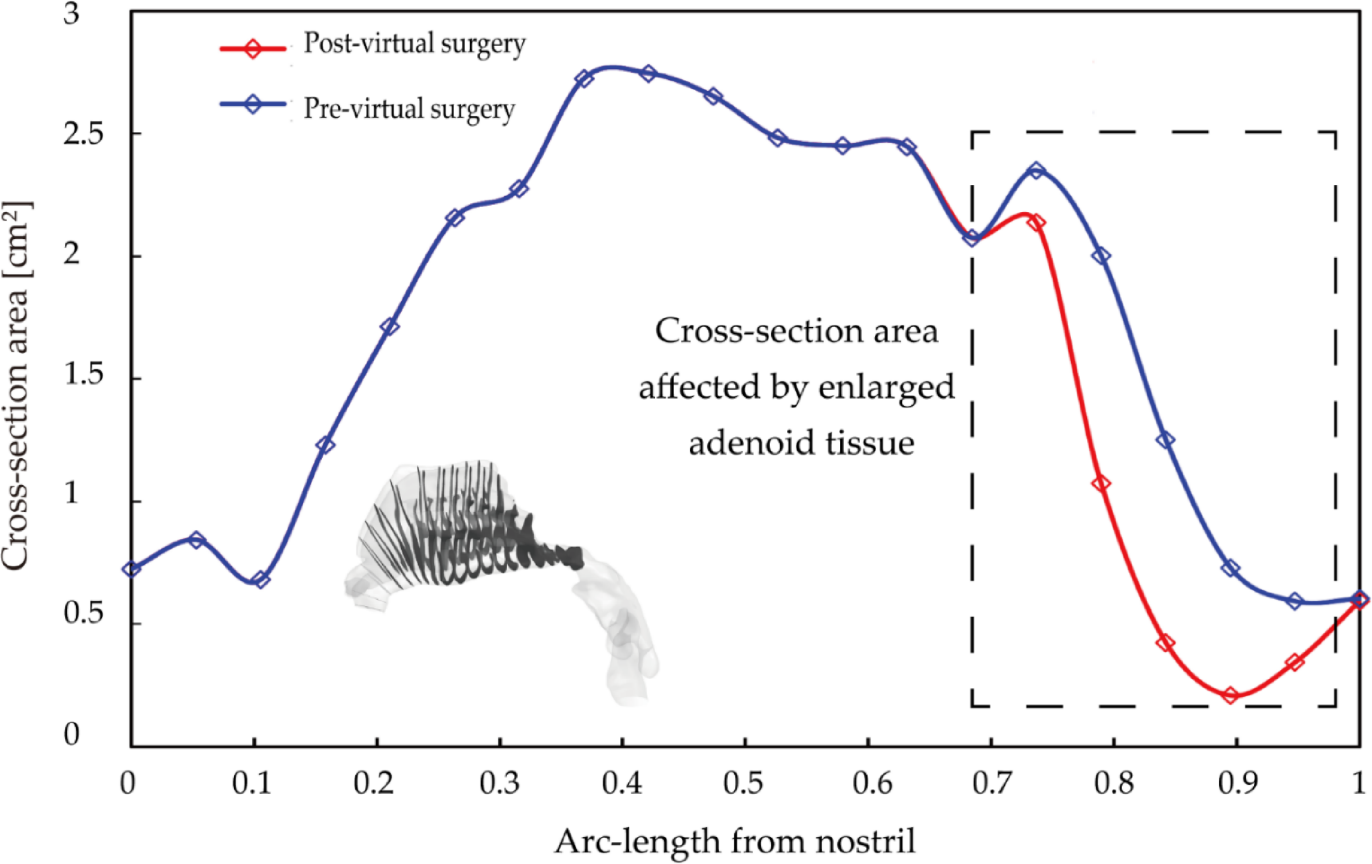
Cross-section area comparison pre- and post-virtual surgery as a function of normalized arc-length away from nostril, where adenoid hypertrophy region highlighted by the rectangle wireframe.

Figure 6 shows cross-sectional area vs. normalized arc-length away from nostril measured pre- and post-virtual surgery. Since virtual operation only conducted at the enlarged adenoid region, the cross-sectional area of the anterior nasal passage (normalized length before 0.7) was remained in same condition. Key anatomical region has been marked with the rectangle wireframe, which covers the whole adenoid area. Within the marked region, the size of the nasopharyngeal channel performs sharp narrow after a moderate increase. The cross-sectional area of the airway after surgery is significantly larger than that of pre surgery, and the difference become greater as the direction away from the nostrils. At normalized distance 0.75, the regional cross-section area reached peak value, 2.2 cm^2^ pre-operation and 2.5 cm^2^ post-operation. After dramatically constriction, the narrowest airway pathway was observed around distance 0.9, which increased from 0.21 cm^2^ to 0.85 cm^2^ after the virtual operation. More than 4-times airway widening brings a non-negligible improvement in nasal airway patency.

### Airflow dynamics

**Figure 7:**
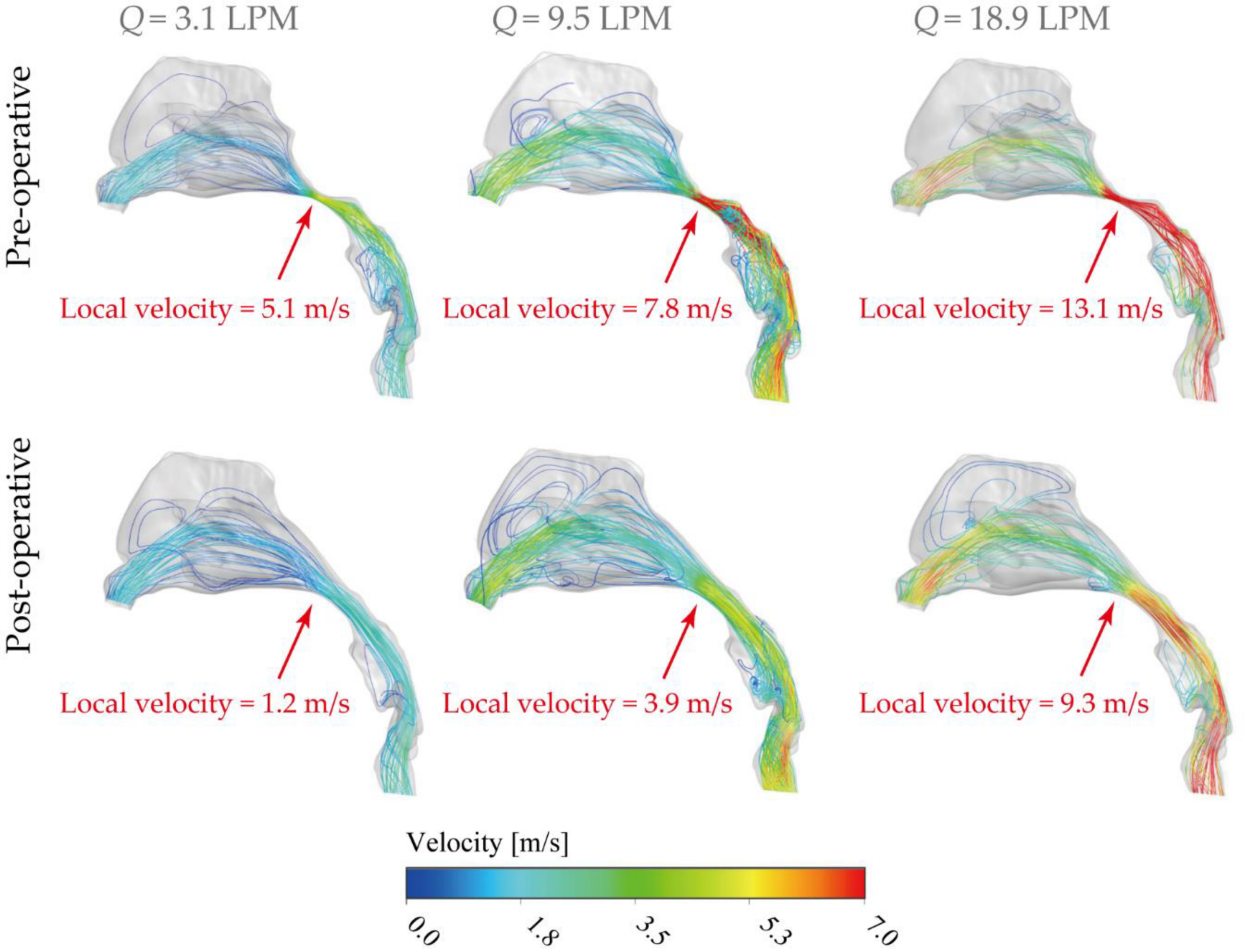
Lateral view of velocity streamlines [m/s] pre- and post-operative under inhalation flow rates of 3.1 LPM, 9.5LPM and 18.9LPM Nasal, with local nasopharyngeal peak velocity highlighted in the figure.

Figure 7 compares velocity streamlines under three inhalation conditions, including 3.1 LPM, 9.5 LPM and 18.9 LPM, which represent different activity levels (resting, light exercise, heavy exercise) before and after virtual surgery. Airflow streamlines analysis provides surgeons with more accurate understanding of the improvement of the nasal airflow after surgery and helps to standard objective judgement of the surgery.

Overall, the flow stream mainly allocated into the middle passage for all inhalation conditions while relatively moderate flow entered the inferior passage. Rare flow reached and formed recirculation stream around the upper anterior site, limited by the vestibule notches and the narrow slit-like anatomical features. As shown in Figure 7, airflow suddenly accelerated at the nasopharyngeal region in the diseased model due to the severely compressed passage. In contrast, the dramatical acceleration situation was significantly alleviated by virtually removing the enlarged tissue, thus more breathing effort is anticipated in diseased nasal cavity to combat the flow resistance while satisfying the respiratory needs. For resting condition (3.1 LPM), local peak velocity of diseased model was measured as 5.1 m/s and it reduced to 1.2 m/s after surgery, which provides the patient with the most noticeable respiratory improvement (more than 4 times) among all activity conditions. Under light exercise condition (9.5 LPM), local peak velocity at nasopharynx region reached 7.8 m/s and the virtual surgery helps to reduce to a half (3.9 m/s). It indicates the operation could achieve considerably satisfying performance in the most common respiratory activity. For heavy exercise condition (18.9 LPM), the extremely high local flow stream was released from 13.1 m/s to 9.3 m/s after virtual surgery. In addition, the distribution of inhaled flow throughout the nasal cavity is more spread out after removing the abnormal tissue since more airflow distributed into olfactory region and superior meatus, which is of great advantage of the nasal ventilation and maintaining stable nasal function.

To further quantify the anatomical effect of the narrowed nasopharynx airway, detailed comparison of pressure drop between original (pre-operative) and virtually restored (post-operative) nasal airways under various inhalation status was performed. As listed in Table 1, the local pressure drop across the nasopharynx region shows obvious variability between pre- and post-operative models. Under all breathing conditions investigated, high pressure drop values were dramatically reduced in the post-operative model, which indicates breathing efforts were reduced and healthier respiratory resistance was restored (46). Among all conditions, the most significant improvement was found under heavy exercise (18.9 LPM), where local pressure drop decreased from 208.66 Pa to 35.52 Pa with an 83% resistance reduction increment.

**Table 1:** Pressure drop (Pa) across the nasopharynx section for pre- and post-operative models under three inhalation conditions: resting (3.1 LPM), light exercise (9.5 LPM), and heavy exercise (18.9 LPM).

	Resting (3.1 LPM)	Light exercise (9.5 LPM)	Heavy exercise (18.9 LPM)
Pre-operative	7.3 Pa	57.14 Pa	208.66 Pa
Post-operative	1.40 Pa	12.7 Pa	35.52 Pa

### Wall shear stress distribution

**Figure 8:**
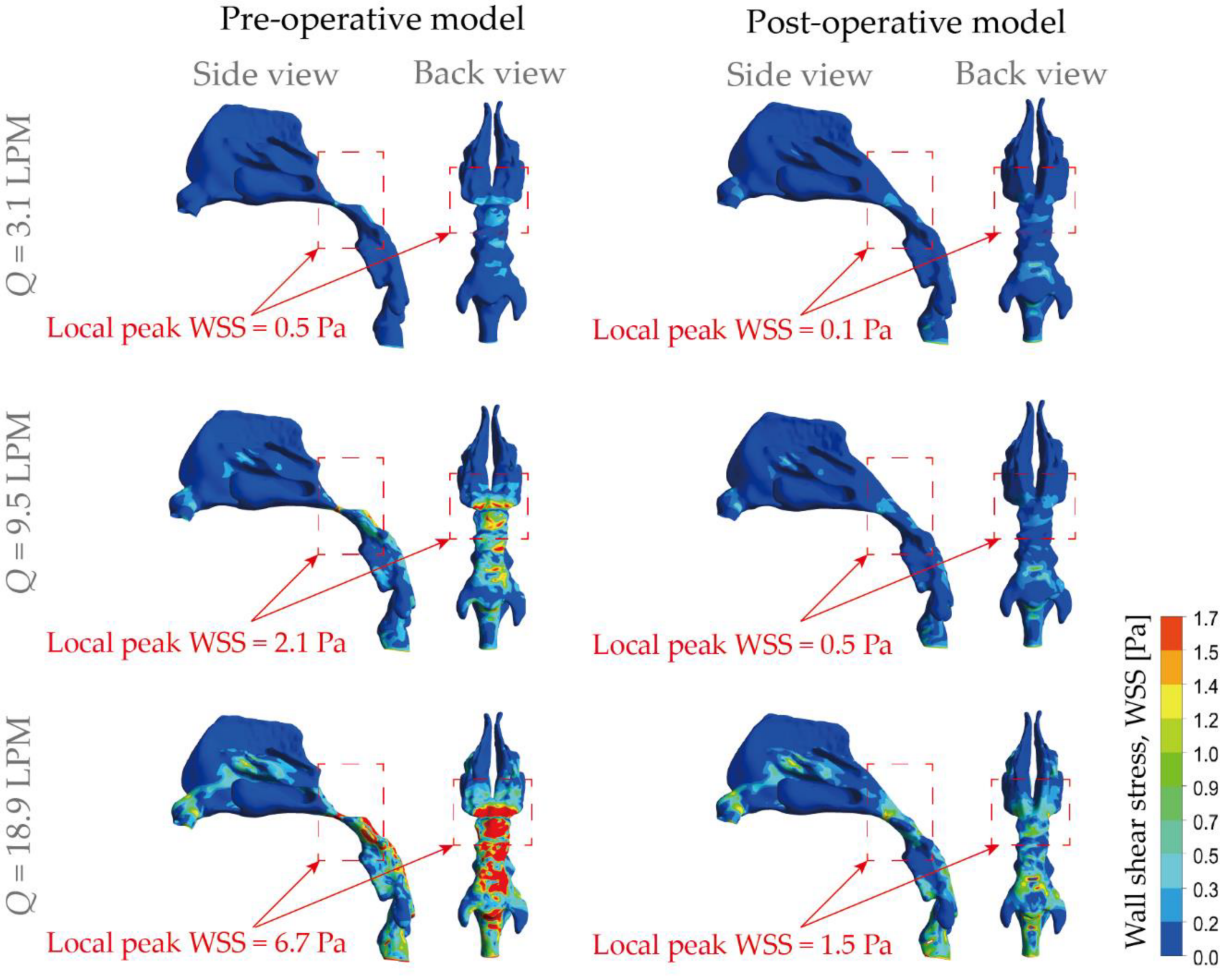
Comparison of wall shear stress (WSS) contours pre- and post-operation, under different inhalation flow rates ranging from 3.1 LPM to 18.9 LPM.

The nasal epithelium is constantly subjected to wall shear stress (WSS) induced by respiratory airflows, and WSS has an important role in the mechanical regulation of the nasal epithelium function. For example, one major source of the mucus secretion is the nasal epithelial goblet cells, which rapidly discharge mucus in response to various biological stimuli. In-vitro cell based assays conducted by Even-Tzur, Kloog (47). has revealed that mucus secretion levels were directly related to the WSS magnitudes and exposure time duration. Abnormal shear stress patterns and prolonged inhalation exposure may lead to permanent cell damage and impaired nasal physiology and defence functions. In this study, WSS at the air-wall interface of the nasal airways were numerically compared between the pre- and post-operation nose subjects (Figure 8). Overall, increased stress occurred at the posterior vestibule region and nasopharynx region due to the 90° turn and constriction of the airway. By the contrary, extremely low magnitude of WSS was observed at the olfactory region and inferior meatus. Specially, it is noticeable that the abnormal enlargement of adenoid tissue caused obviously varying stress pattern that has been marked by red rectangle in Figure 8. For resting breathing condition (3.1 LPM), shear stress at nasopharynx region exhibits slight increase with peak value of 0.5 Pa while 0.07 Pa was measured after virtual surgery which only accounts 14% of the original case. For light exercise condition (9.5 LPM), abnormally high WSS was observed for diseased model with local peak magnitude of 2.1 Pa, and the value reduce to a quarter after virtual surgery. Extremely high WSS was prevalent in marked region in diseased model under active inhalation situation with local peak of 6.7 Pa and it reduced to 22% of diseased case. Obvious relief of WSS variation support patient maintain more stable nasal function under active situation as less stimulation is imposed. In addition, relatively increased stress exhibits in the middle passage since rapid near-wall velocity variation owing to fast flow stream passing through.

### Regional flow distribution

In order to accurately analyze the improvement of airflow distribution by the virtual surgery, a cross-sectional slice was taken in the middle passage, where the middle and inferior meatus were divided and statistical volume flow for pre- and post- surgery is shown in Table 2 [Improvements in airflow distribution are measured using increments: (post-surgery—pre-surgery)/pre-surgery × 100]. It can be observed that the increment of the inferior meatus post-surgery is much larger than that of the middle meatus. From the perspective of data analysis, the virtual surgery has a stronger effect on the improvement of the airflow into the inferior meatus, which is due to the smaller value of the measured volume flow. For heavy exercise (18.9 LPM), the passing flow increases most, reaching 28.7%. However, in various breathing states, the amount of airflow entering the middle meatus is significantly more than that of the inferior meatus, showing a law that increases with the higher inhalation rate. When the child was breathing vigorously, the most abundant flow through middle meatus increased from 11.15 m^3^/s to 12.11 m^3^/s, with an increment of 8.61%. The improvement in virtual surgery is most pronounced in the light exercise condition (9.5 LPM), because 11.05% more flow was apportioned into the middle meatus.

**Table 2:** Comparison of local flow distribution* in the middle and inferior meatus between pre- and post-virtual surgery subjects.

Inhalation flow rate	Resting (3.1 LPM)			Light exercise (9.5 LPM)			Heavy exercise (18.9 LPM)		
Case studies	Pre-	Post-	Increment*	Pre-	Post-	Increment*	Pre-	Post-	Increment*
Middle meatus	1.63	1.64	0.42%	5.62	6.24	11.05%	11.15	12.11	8.61%
Inferior meatus	0.07	0.10	27.27%	0.06	0.08	27.67%	0.26	0.33	28.70%

*Measurement plane was taken at the middle of nasal cavity. All localized flow distribution of case “Pre-” and “Post-” are in the unit of cm^3^/s.

*Increment = 100% × (post-surgery—pre-surgery)/pre-surgery.

### Olfactory ventilation

**Figure 9:**
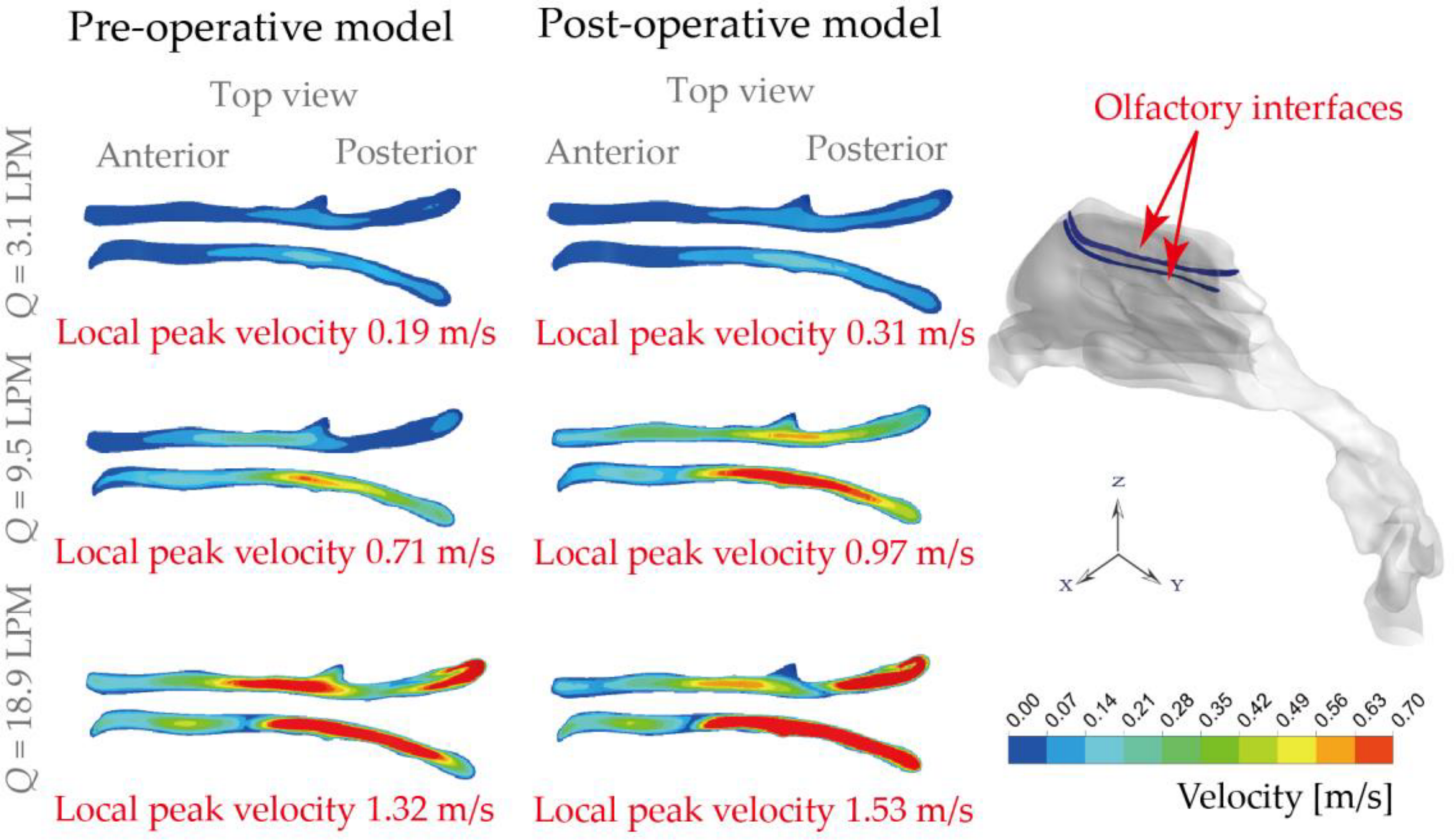
Olfactory ventilation comparison based on local velocity distributions at the olfactory interfaces, under inhalation flow rate of 3.1 LPM, 9.5 LPM and 18.9 LPM.

Nasal airflow that effectively transports ambient odours to the olfactory receptors is important for human olfaction. On the other hand, the olfactory region itself is a portal for intranasal drug aerosol delivery that targets the Central Nervous System (CNS). To evaluate the anatomical changes (i.e., normal and obstructed nasopharynx region due to the presence of adenoid hypertrophy) on airflow that enters the olfactory slits, the ventilation status of pre- and post-operation nose subjects were numerically compared (Figure 9). Overall, weak flow enters olfactory region and the situation shows limited improvement with more active respiratory conditions. Under 3.1 LPM breathing condition, rarely gentle flow entered olfactory region in original model with a local velocity of 0.19 m/s and it is improved 160% after virtual operation. For general breathing condition (9.5 LPM), relatively limited enters olfactory slit before operation with the local peak velocity of 0.71 m/s while inter-chamber difference is slightly eliminated with local velocity increasing by 136% after surgery. Compared with gentle and general condition, relatively effective flux flows into that region with measured local velocity of 1.32 m/s which rises to 1.53 m/s after operation. The weak improvement exhibited under active respiratory condition was owing to the anatomical limitation effect.

### Particle deposition analysis

**Figure 10:**
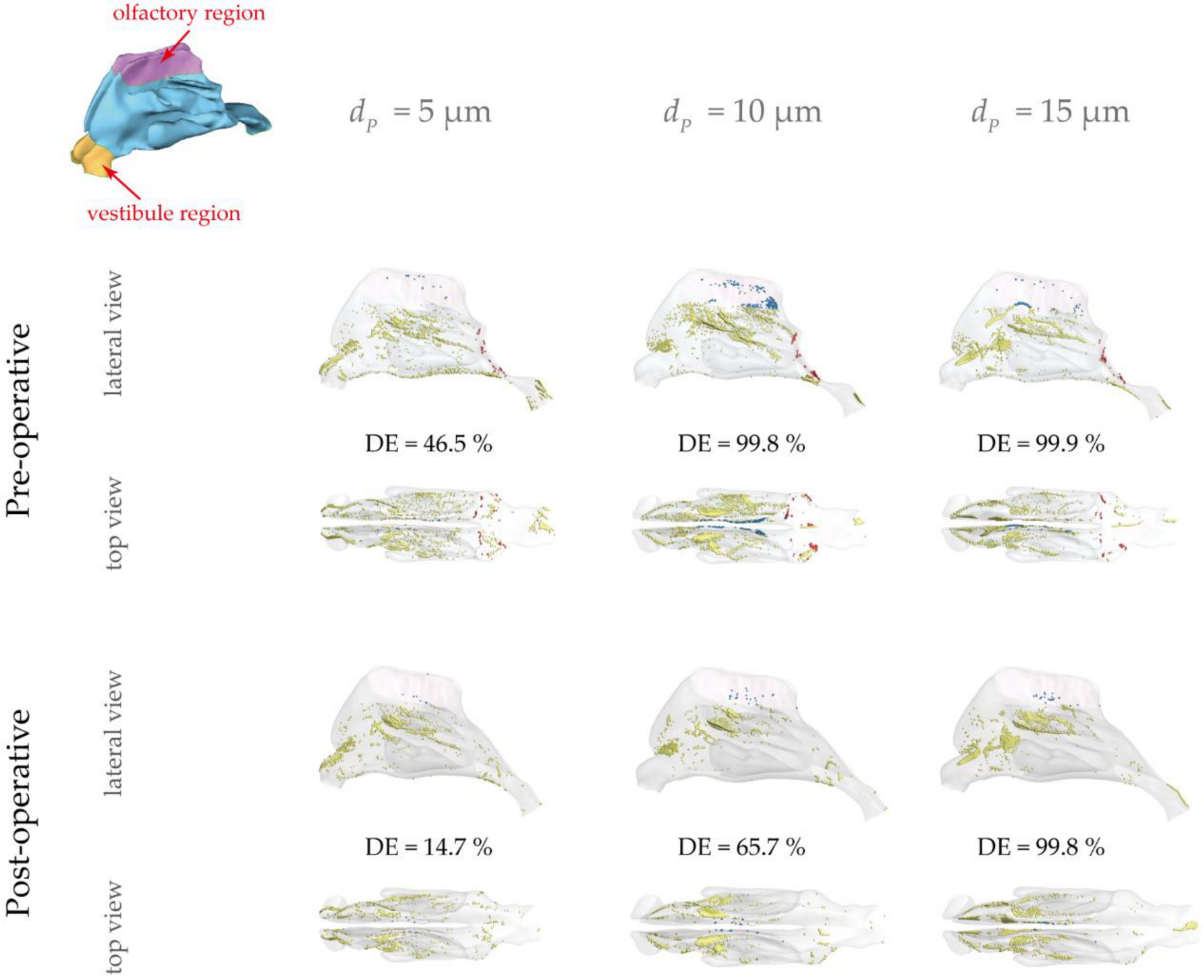
Micro-particle deposition patterns under 9.5 LPM pre- and post-operation with a particle diameter of 5 *μ*m, 10 *μ*m and 15 *μ*m. Patterns are colored based on particle deposition region and those trapped at the abnormal adenoid surface are highlighted by blue. Nasal surface is shown with 10% transparency.

Figure 10 describes micro-particle (with diameter of 5, 10, 15 μm) deposition patterns under the light exercise inhalation condition (9.5 LPM), from which noticeable variation of the spatial deposition performance is observed between pre- and post-surgery models. Overall, higher nasal deposition intensity appeared for larger particle size because of the inertia-dominated deposition mechanism. Surface deposition for 5 μm exhibited a relatively dispersed pattern while that for other two particle sizes were more concentrated at the middle passage since the large inertia particles were more likely to transport along the inhaled flow and hit nasal surface. Similarly, the larger the particle inertia, the higher deposition distribution at anterior passage. There was rare deposition spot being observed in the inferior and upper passage since limited flow distribution here. However, 10 μm showed the most concentrated pattern in the olfactory region compared to the 5 μm and 15 μm.

**Figure 11:**
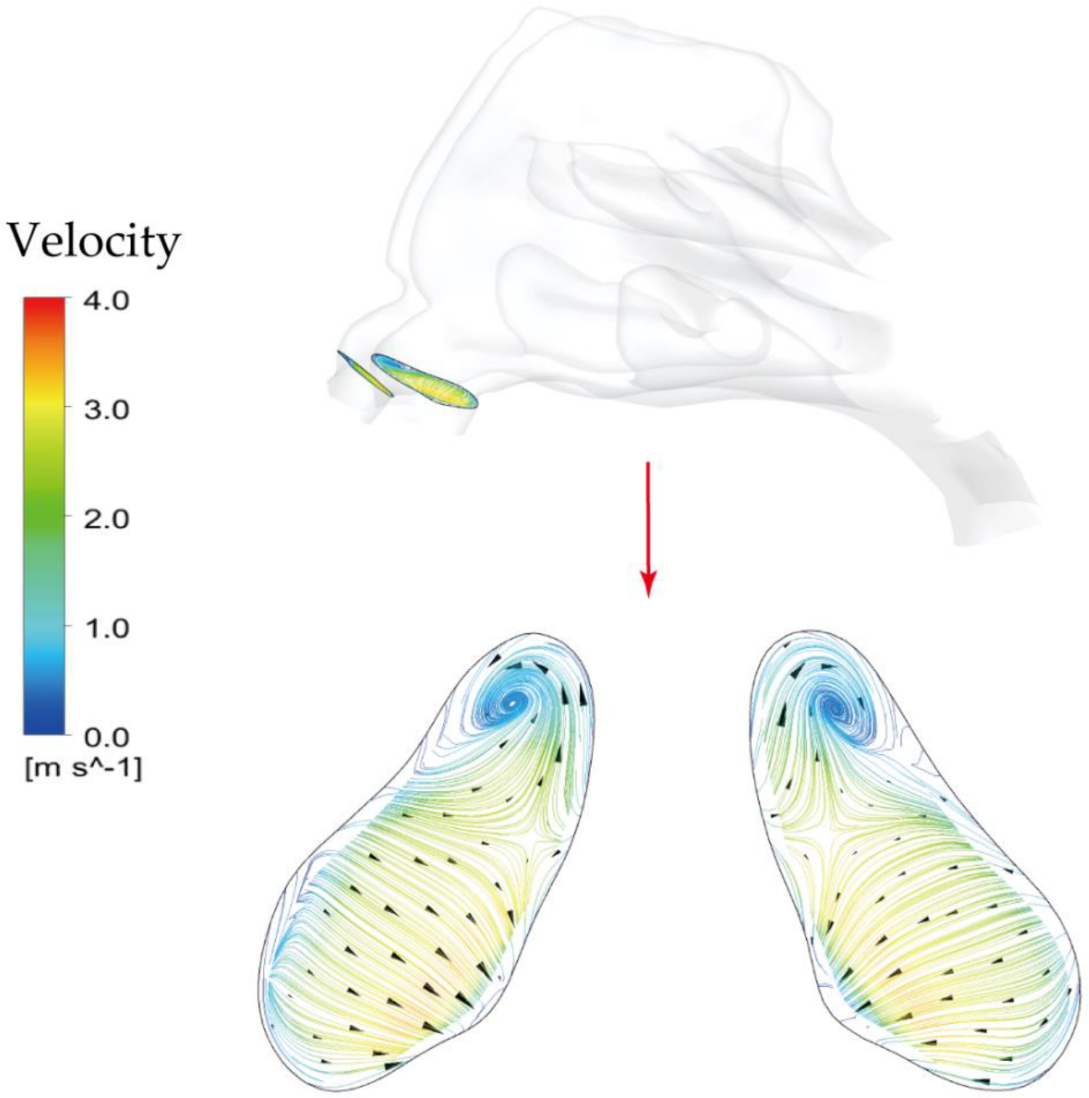
Secondary flow patterns on a cross-sectional plane in the middle of vestibule region under light exercise inhalation condition (9.5 LPM).

It is observed that 5 μm is preferred to deposit in the vestibule region while 10 μm and 15 μm were more likely to escape from the filtration of this region and direct into main passage. The localized deposition behaviour could be characterized by Stokes number which is considered as a significant non-dimensional parameter for describing particle motion (48). It suggests that particles become increasingly sensitive to the flow with smaller Stokes numbers. Particles of 5 μm has smallest Stokes number that is proportional to the square of the particle diameter, resulting in the highest fidelity of flow tracer behaviour. Figure 11 describes the secondary flow motion on a cross-sectional plane in vestibule region, from where discernible vortices is observed at the top of plane due to the local airway changes. Consequently, particles of 5 μm are most likely to comply the local flow changes and hit onto the wall, forming the most concentrated deposition at the roof top of vestibule region. This unique finding may distinguish from other literature that reported higher deposition efficiency for larger micron-particles in vestibule region by the different definition of vestibule region and inter-subject anatomical feature of nasal vestibule (19, 49). It was more commonly that defining the entrance and nasal valve as vestibule, which provides the deposition results that measured within a quite larger area than this study. Furthermore, the cylindrical shape of children nasal vestibule and the nearly parallel cross-sections of nostrils and nasal valves facilitate extensive contact between inhaled flow and vestibule surface, enhancing the possibility of airflow responding to local airway changes. This encourages that there is still valuable research potential in the morphological features of children nasal cavity and its associated flow and aerosol dynamics.

For all particle sizes, pre-surgery nasal geometry shows higher possibility of capturing micron-particles compared with post-surgery. In addition, deposition patterns for pre-surgery model spread further into the posterior passage compared to post-surgery model, which may owe to a relatively plain flow jet path from nostril to pharynx that formed by severely blocked nasopharynx. Besides, more particles tended to deposit into olfactory region pre-surgery, which was particularly obvious for 10 μm particles. This funding may provide more evidence to help the nasal-brain related drug delivery research.

Furthermore, it needs to be noted that the deposition pattern and regional disparities were discussed based on the assumption of constant flow rate under 9.5 LPM, which neglects the instantaneous deposition of microparticles because of unsteady accelerating and decelerating flow. For instance, overprediction of deposition in anterior nasal passage was reported under steady condition when particle size is between 1 and 20 μm (50). A higher deposition with particle sized between 1 and 10 μm for steady condition was found by Häußermann, Bailey (51). However, our previous study also demonstrated that the quansi-steady airflow assumption in the nasal cavity was reasonable when the instantaneous Strouhal number was smaller than 0.2 (52). Therefore, in present study, the instantaneous deposition features were not considered, and future studies should consider addressing this research limitation.

**Figure 12:**
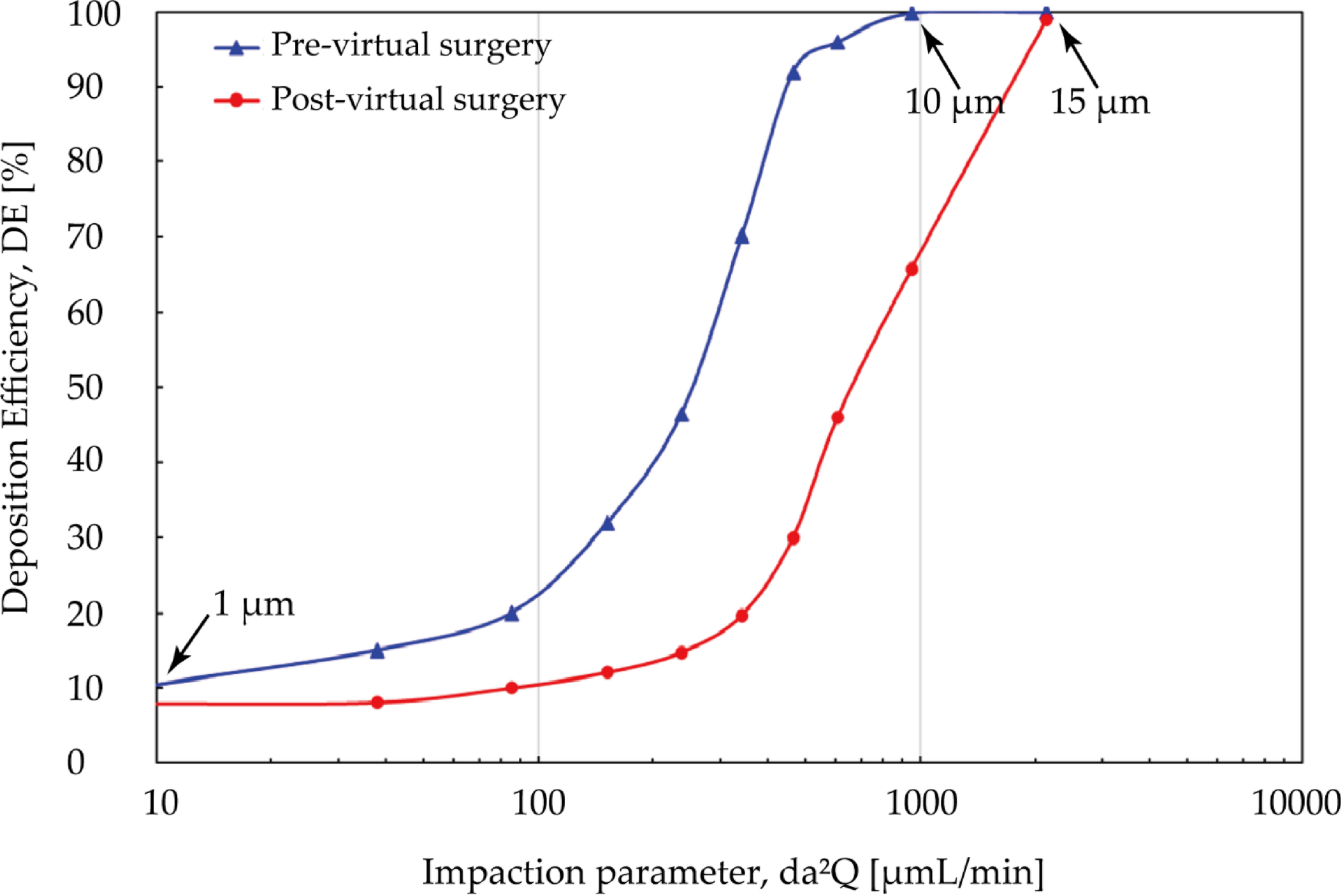
Comparison of total deposition efficiency [%] for pre- and post-surgery case vs. impaction parameter under inhalation flow rate of 9.5 L/min. The particle diameter is with a range from 1 μm to 30 μm.

Figure 12 measured total nasal deposition efficiency (DE) and clearly present the contrast between pre- and post- virtual surgery vs. impact factor. For the overall trend, the number of particles captured by the nasal surface wall was significantly positively correlated with the impact factor, and DE increased at a greater rate when the particle diameter exceeded about 4 μm. Before the virtual operation, DE gradually approaches 99.9% from lower levels (10.2%) with increasing particle diameter up to 10 μm. Comparing the results before and after surgery, total nasal deposition of each particle diameter is reduced to varying degrees. The higher velocity streamlines resulting from the narrow airway before surgery makes inertial-dominated transport of micro-sized particles more easily captured by the complex nasal structure. The deposition curves exhibit greater difference (approximately 3.2 times difference) when inhaled particle size larger than 5 μm and the curve approaches peak until 15 μm.

**Figure 13:**
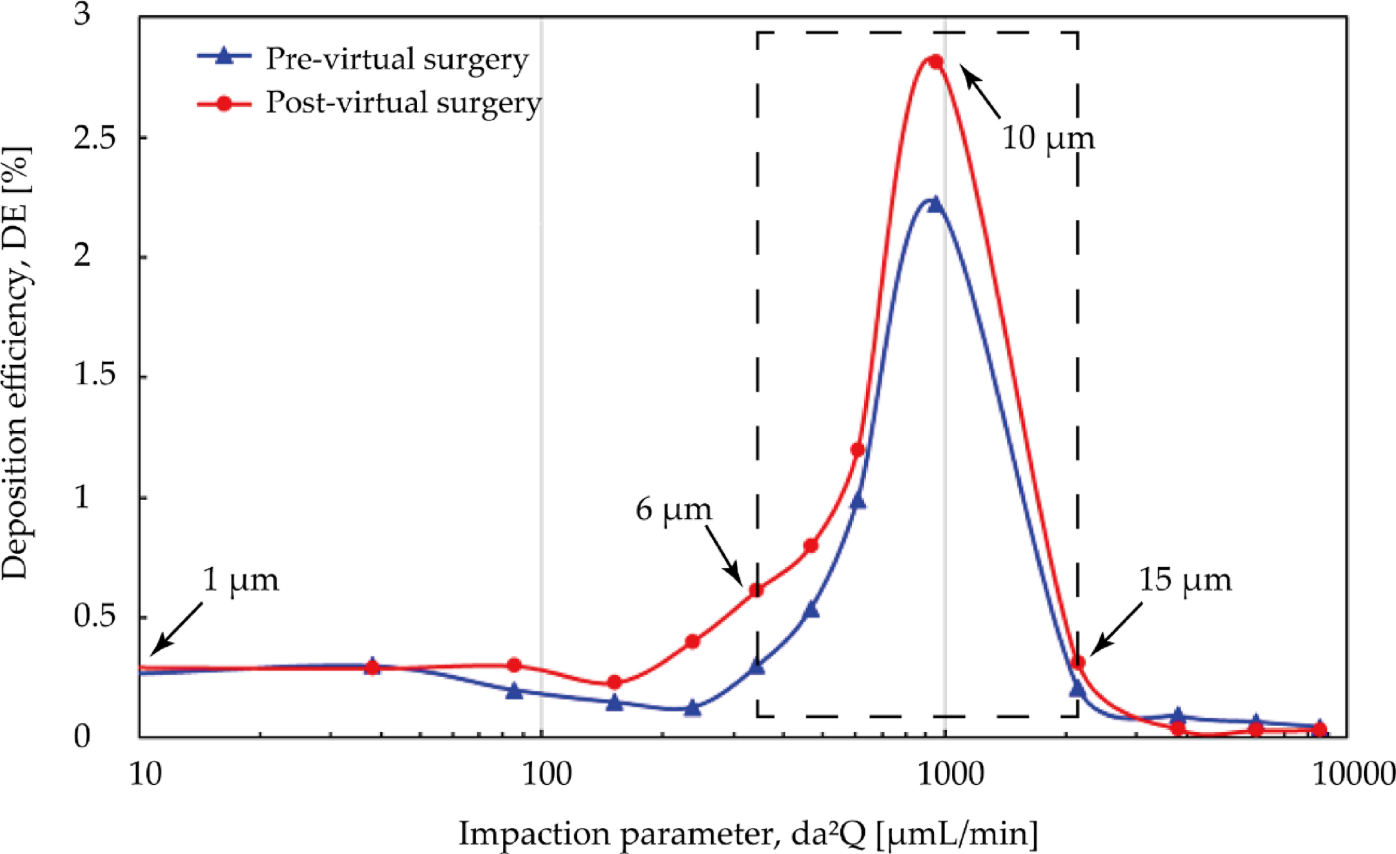
Comparison of regional deposition efficiency in adenoid hypertrophy area [%] for pre- and post-surgery case vs. impact factor under inhalation flow rate of 9.5 L/min. High deposition efficiency range at 6 μm and 15 μm are marked with black arrows.

Although adenoidectomy is the most common treatment for severe cases, a few studies indicated that using nasal steroids including budesonide, mometasone furoate is also considered as an effective therapeutic method for adenoid hypertrophy treatment specially for decreasing diseased tissue before surgery or preventing regrowth after surgery (27, 40, 53). Several possibilities have been raised about its mechanism, including a lympholytic action on adenoids, anti-inflammatory effect, or reduction the risk of the adenoids for infection. Since intranasal steroids is considered as a therapeutic treatment or adjunctive treatment after surgery, the adenoid region that lesion occurs should be paid attention to as the destination for drug delivery. Figure 13 is comparison of regional DE in adenoid tissue area for pre- and post- virtual surgery vs. impact factor (for convenience of observation, the value range of the ordinate becomes 0 to 3). Overall, similar lower deposition results occurs when particle diameter smaller than 5 μm or larger than 15 μm while peak-shaped deposition curves appear in the middle of this range (reaches peak at 10 μm). It is worth noting that DE measured after virtual adenectomy is higher than that before virtual surgery, especially when particle diameter is 10 μm. The enlarged adenoids tissue forms a locally high-speed airflow and jet to the back of the pharynx, so that a more linear streamline transports the particles through the target area.

## Conclusions

This study systematically compared changes in anatomical morphology, airflow dynamics, and aerosol drug delivery features of a three-year-old children nasal cavity who diagnosed with adenoid hypertrophy for pre- and post-virtual adenectomy. Ultrafine particles ranged between 1 and 20 μm were simulated to evaluate the potential drug aerosol delivery characteristics in nasal subjects before and after virtual adenoid adenectomy. In detail, virtual surgery removed redundant part of the diseased adenoid which obstructed 75% of the nasopharyngeal passage, which expands the local airway lumen size up to 4 times. For the main nasal passage section, airflow predominantly enters the middle meatus than that of the inferior meatus in all considered breathing conditions. For the obstructed nasopharynx section, the restored airway anatomy greatly alters the airflow characteristics and resultant wall shear stress distribution. Specifically, for light exercise breathing condition (*Q* = 9.5 LPM), the peak velocity at nasopharyngeal region was reduced from 7.8 m/s to 3.9 m/s in the post-operational model. Similar changes were found in wall shear stress distributions, and local peak value was reduced from 2.1 Pa to 0.5 Pa, which is in line with stress levels observed in healthy normal airways (54). In summary, the unimpeded internal nasopharynx region with diseased tissue removed significantly reduces trans-nasal pressure drop by up to 83%, which substantially reduces the patient's effort to maintain general respiratory activities. Meanwhile, for the same breathing condition, better local ventilation was also observed in the olfactory slits, the resultant local ventilation rate is 1.36 times stronger than that in the obstructed nasal subject. For passively released drug aerosols, despite the post-surgery nasal passage exhibited lower total deposition efficiency compared with its original obstructed nasal airway, more concentrated regional deposition was observed in the nasopharynx area adjacent to the adenoid. Regional deposition improvements were observed in this area for particles ranging between 6 and 15 µm, which may of important relevance to the postoperative medication treatment and nasal drug therapy for adenoid hypertrophy.

In summary, the reduced airflow velocity, wall shear stress at the affected area, the improved local ventilation in olfactory slits, as well as the greatly mitigated flow resistance due to the expanded airway and the removal of flow disturbance, all contribute to healthy epithelium cell exposure at the air-wall interface and improved nose physiology, which could ultimately lead to better quality of daily life for patients. Furthermore, the improved aerosol delivery at affected region may facilitate to postoperative inhaled medication treatment for adenoid hypertrophy.

One major research limitation of present study is the limited nasal airway subject. Apparently, the factor of inter-subject variation on airflow and associated particle deposition characteristics cannot be considered based on only one nasal airway subject. Additionally, the steady flow assumption represents another research limitation for this research, where the unsteadiness of the inspiratory airflow is neglected. Despite existing research limitations, the present numerical pre- and post-operative models and research fundings still provide scientific evidence to children adenoid hypertrophy examination and present reference towards optimal patient-specific treatment outcomes.

## Data Availability

The original contributions presented in the study are included in the article/**Supplementary Material**, further inquiries can be directed to the corresponding author/s.
