# Supplementary material for: Numerical modelling of micron particle inhalation in a realistic nasal airway with pediatric adenoid hypertrophy: A virtual comparison between pre- and postoperative models
Source: Front Pediatr. 2023 Feb 23;11:1083699. doi: 10.3389/fped.2023.1083699 (PMC9996336; doi:10.3389/fped.2023.1083699)
Supplement: Supplementary file 1 [file Datasheet1.pdf]

Inhalation rate(LPM)	18.9			9.5			5		
Case	Pre-	Post-	Increment (%)	Pre-	Post-	Increment (%)	Pre-	Post-	Increment (%)
Middle meatus	12.11	11.15	8.61	6.24	5.62	11.05	1.64	1.63	0.42
Inferior meatus	0.33	0.26	28.70	0.08	0.06	27.67	0.10	0.07	27.27

Table 1. Comparison of flow distribution difference in the middle and inferior meatus before and after the virtual surgery.

*Measurement plane was taken at the middle of nasal cavity

*Increment(%)=(post-surgery - pre-surgery) /pre-surgery×100)
